# Overall side‐effect bother consistently associated with early treatment discontinuation due to adverse events in four clinical trials with various cancer types and treatments

**DOI:** 10.1002/cncr.70084

**Published:** 2025-10-22

**Authors:** John Devin Peipert, Shaili Ganatra, Fengmin Zhao, Ju‐Whei Lee, Jessica Roydhouse, Edward H. Ip, Nathaniel O’Connell, Ruth C. Carlos, Noah Graham, Mary Lou Smith, Ilana F. Gareen, Ahmad A. Tarhini, Tait Shanafelt, Vered Stearns, Pamela J. Raper, David Cella, Robert Gray, Lynne I. Wagner

**Affiliations:** ^1^ Centre for Patient Reported Outcomes Research Department of Applied Health Sciences University of Birmingham Edgbaston Birmingham UK; ^2^ Department of Medical Social Sciences Feinberg School of Medicine Chicago Illinois USA; ^3^ Eastern Cooperative Oncology Group‐American College of Radiology Imaging Network Biostatistics Center Dana‐Farber Cancer Institute Boston Massachusetts USA; ^4^ Menzies Institute for Medical Research University of Tasmania Hobart Tasmania Australia; ^5^ Department of Biostatistics and Data Science Wake Forest University School of Medicine Winston‐Salem North Carolina USA; ^6^ Department of Radiology Columbia University Vagelos College of Physicians and Surgeons New York New York USA; ^7^ Research Advocacy Network Chicago Illinois USA; ^8^ Department of Epidemiology and the Center for Statistical Sciences Brown University School of Public Health Providence Rhode Island USA; ^9^ Department of Oncologic Sciences H. Lee Moffitt Cancer Center Research Institute University of South Florida Morsani College of Medicine Tampa Florida USA; ^10^ Department of Internal Medicine Stanford University School of Medicine Stanford California USA; ^11^ Department of Medicine Division of Hematology and Medical Oncology Weill Cornell Medicine New York New York USA; ^12^ Department of Health Policy and Management Gillings School of Global Public Health University of North Carolina at Chapel Hill Chapel Hill North Carolina USA

**Keywords:** clinical trials, patient‐reported outcomes, quality of life, tolerability

## Abstract

**Background:**

Overall side‐effect impact, the aggregated experience of multiple toxicities, can be captured with the single Functional Assessment of Cancer Therapy (FACT) item GP5 (“I am bothered by side effects of treatment”). The objective of this study was to evaluate the ability of item GP5 to indicate treatment tolerability by examining its association with early treatment discontinuation (ETD) due to adverse events (AEs) in four cancer trials.

**Methods:**

The authors analyzed data from four clinical trials coordinated by the ECOG‐ACRIN Cancer Research Group, covering breast cancer (E1Z11, all patients received anastrozole; E1Z03, compared anastrozole vs. exemestane), melanoma (E1609, compared ipilimumab vs. high‐dose interferon α), and chronic lymphocytic leukemia (E1912, compared ibrutinib–rituximab bs. standard chemoimmunotherapy). In each trial, GP5 responses were categorized as severe bother (i.e., “very much”/“quite a bit”) versus moderate/low bother (“somewhat”/“a little bit”/“not at all”).

**Results:**

At 3 months, significant associations were observed in each trial, indicating approximately 2.8–6.8 times greater odds of ETD among patients reporting severe bother (E1Z03: adjusted odds ratio [aOR], 2.82; 95% confidence interval [95% CI], 1.10–7.28; E1Z11: aOR, 4.10; 95% CI, 1.69–10.00; E1609: aOR, 6.77; 95% CI, 2.88–15.92; E1912: aOR, 4.15; 95% CI, 1.64–10.49).

**Conclusions:**

These results support prior research demonstrating an association between severe side‐effect bother, as reported on responses to the GP5 item, and ETD among patients with newly diagnosed multiple myeloma. The consistent association between GP5 responses and ETD suggests the GP5's ability to capture how well patients tolerate treatment, especially when side‐effect bother is observed early in the course of treatment, and signals an opportunity to develop interventions to promote treatment adherence.

## Abstract 2

A brief, patient‐reported rating of overall side‐effect bother, the FACT GP5, was associated with a greater risk of early treatment discontinuation in multiple clinical trials. This brief patient report signals a promising way to assess the tolerability of cancer treatments.

## INTRODUCTION

There is increasing recognition of the importance of patient report in capturing cancer treatment tolerability. Symptomatic adverse events (AEs) are frequently underreported by clinicians,^1^ indicating that the most valid approach to their assessment is directly from patients. With this in mind, in 2018, the Friends of Cancer Research convened experts to put forth an updated definition of treatment tolerability that centers patient‐reported outcomes (PROs).^2^ The US Food and Drug Administration (FDA) has also advanced a core set of PROs for use in cancer trials that includes multiple tolerability‐related PRO concepts.^3^ Complementing and contemporary to this work was the activity of the US National Cancer Institute (NCI) Cancer Treatment Tolerability Consortium.^4^ This consortium includes four research teams and representatives from the NCI and the FDA focusing on advancing different approaches and aspects of capturing treatment tolerability using PROs.

Both the NCI Cancer Treatment Tolerability Consortium and the FDA have focused extensively on the concept of overall side‐effect impact or bother.^4^, ^5^ The FDA's publication *Core Patient‐Reported Outcomes in Cancer Clinical Trials: Guidance for Industry* points to the value of summarizing the impact of multiple different side effects on a single patient.^3^ In addition, an overall summary measure may facilitate comparison of tolerability of two or more treatments with different side‐effect profiles. To date, the primary way to measure overall side‐effect impact has been the single Functional Assessment of Cancer Treatment (FACT) GP5 item (GP5), “I am bothered by side effects of treatment,” with response options ranging from 0 (“not at all”) to 4 “(very much”). The GP5 item has been the subject of several recent studies to examine its validity and usefulness.^6^, ^7^, ^8^, ^9^, ^10^

In addition to psychometric evaluation studies, there has been interest in the degree to which GP5 is associated with subsequent early treatment discontinuation, a common indicator of treatment tolerability. Wagner et al. observed that GP5, when measured at baseline, was a significant predictor of early treatment discontinuation in the ECOG‐ACRIN E1Z03 trial among postmenopausal women with hormone receptor‐positive, primary, invasive breast cancer.^11^ Although the study by Wagner et al. was a useful evaluation of GP5's potential as a screener for early treatment discontinuation, more research was needed to determine how GP5 reflected patients' experience of side effects while on treatment. A first study to address this question indicated that GP5 captured postbaseline in the ECOG‐ACRIN E1A11 multiple myeloma trial was associated with 2.0–4.5 times greater odds of early treatment discontinuation due to AEs.^12^ In the current study, we sought to evaluate the generalizability of these findings by analyzing additional clinical trials with differing cancer diagnoses and treatments.

## MATERIALS AND METHODS

### Clinical trial data sets

#### ECOG‐ACRIN E1Z11

Trial E1Z11 (ClinicalTrials.gov identifier NCT01824836), a cohort study to evaluate genetic predictors of aromatase inhibitor musculoskeletal symptoms, enrolled postmenopausal women who were receiving aromatase‐inhibitor therapy for stage I–III, hormone receptor‐positive, early breast cancer.^13^ Participants with an ECOG performance status of 0–2, a pain score of 0–3 of 10, and no rheumatologic comorbidities initiated open‐label anastrozole. Participants received anastrozole for 12 months. The primary end point was discontinuation of treatment because of musculoskeletal symptoms, as rated using the Health Assessment Questionnaire. Secondary end points were measured using multiple PROs, including the FACT‐Endocrine Symptoms instrument, which includes the GP5 item and was administered at baseline and at 3, 6, 9, and 12 months.

#### ECOG‐ACRIN E1Z03

Trial E1Z03 trial (ClinicalTrials.gov identifier NCT00090974) evaluated treatment side effects associated with anastrozole or exemestane among postmenopausal women with receptor‐positive, primary breast cancer who were enrolled in the MA.27 trial.^11^ Treatment was offered for up to 5 years. Participants in the Canadian Clinical Trials Group MA.27 trial (ClinicalTrials.gov identifier NCT00066573) were adults with an ECOG performance status of 0–2 who had no previous cancer treatment or a washout after previous treatment. The primary end point was the overall response rate, and secondary end points included progression‐free survival and overall survival between treatment arms.^14^ E1Z03 participants completed the FACT‐Endocrine Symptoms instrument (including the GP5 item) at baseline and at 3, 6, 12, and 24 months.

#### ECOG‐ACRIN E1609

Trial E1609 (ClinicalTrials.gov identifier NCT01274338) was a phase 3 trial comparing the efficacy and safety of adjuvant ipilimumab at two different doses (3 and 10 mg/kg) versus high‐dose interferon alfa‐2b in patients with high‐risk, stage III–IV melanoma that had been surgically removed.^15^ Ipilimumab was administered every 3 weeks for four doses as induction therapy, followed by up to four more doses every 12 weeks. Interferon alfa‐2b was given 5 days per week for 4 weeks, followed by administration every other day, 3 weeks apart, for up to 48 weeks. The total possible time on treatment was 60 weeks for ipilimumab or 52 weeks with interferon alpha‐2b. Participants were adults with an ECOG performance status of 0 or 1 who were within 84 days of last surgery and had no prior systemic adjuvant therapy for melanoma. Primary end points were recurrence‐free and overall survival. Secondary end points included the FACT‐Biological Response Modifiers, which includes the GP5 item and was administered at baseline and every 3 months for up to 15 months.^16^

#### ECOG‐ACRIN E1912

Trial E1912 (ClinicalTrials.gov identifier NCT02048813) was a phase 3 trial comparing the efficacy of ibrutinib (daily until disease progression) plus six cycles of rituximab versus the standard chemoimmunotherapy regimen of fludarabine, cyclophosphamide, and rituximab.^17^ Those randomized to receive ibrutinib plus rituximab did so for seven 28‐day cycles, then received ibrutinib alone until they developed progression or unacceptable toxicity. Those receiving standard chemotherapy did so for six 28‐day cycles. Participants included individuals aged 70 years and younger with previously untreated chronic lymphocytic leukemia or small lymphocytic lymphoma. The primary end point was progression‐free survival, and secondary end points included overall survival and PROs to help capture treatment tolerability. This included the FACT‐Leukemia instrument, which includes the GP5 item and was administered at baseline and at 3, 6, 12, 18, 24, 30, and 36 months.

### Measures

In the current study, the primary independent variable of interest was the GP5 response at 3 and 6 months. We dichotomized GP5 responses in two ways for analyses. First, using a method employed in previous studies,^6^, ^12^ we categorized responses as severe side‐effect bother (responses of “very much” [4] and “quite a bit” [3]) and moderate‐to‐low bother (responses of “somewhat” [2], “a little bit” [1], or “not at all” [0]). Second, we categorized responses as severe‐to‐moderate bother (“very much” to “somewhat”) and low bother (“a little bit” and “not at all”).

The dependent variable of interest was early treatment discontinuation due to AEs. According to each study’s protocols, reasons for treatment discontinuation are recorded in the off‐treatment form. In addition to AEs, other predefined reasons for early discontinuation included disease progression, patient withdrawal or refusal, alternative therapy, death, other complicating disease, and other. To create the study outcome, for each trial, patients for whom the off‐treatment reason was an AE were coded “yes” before 3 months and at 6 months (separately), and those with other reasons or who did not discontinue early were coded “no.” Each patient was classified as having discontinued early either because of AEs or not (including those who discontinued early for other reasons and those who completed therapy according to the protocol). In addition, we included the following patient characteristics: age (years), baseline ECOG performance status (0, fully active; 1, restricted in strenuous activity but ambulatory and able to carry out light work; 2, requires bed rest for <50% of waking hours, cannot carry out work, but can perform self‐care), sex (female, male), and race (American Indian or Alaska Native, Asian, Black or African American, White, and unknown).

### Statistical analyses

Analyses included trial participants who had nonmissing GP5 data at baseline, 3 months, or 6 months. All statistical tests considered a nominal two‐sided *p* value <.05 or a 95% confidence interval (CI) that did not exceed 1 to be statistically significant. All analyses used complete cases only and were conducted in SAS version 9.4 (SAS Institute Inc.). Patient characteristics were summarized with frequencies and percentages or means, standard deviations, and ranges. GP5 response distributions were visualized with a stacked bar chart grouped by clinical trial.

To examine the association between GP5 responses while on treatment and early treatment discontinuation due to AEs, we fit logistic regression models. Each trial was analyzed separately with treatment arms aggregated, and we fit separate models for the 3‐month and 6‐month timepoints. For all models, we included only patients who completed treatment up to the follow‐up time in question (e.g., for the association between GP5 at 3 months and early treatment discontinuation due to AEs, only patients who completed treatment at 3 months were included). For analyses, we omitted patients who reported “very much” on GP5 at baseline because they were already at or near the ceiling for side‐effect bother (E1Z03, *n* = 7 [1.0%]; E1Z11, *n* = 8 [0.8%]; E1609, *n* = 5 [0.9%]; E1912, *n* = 1 [0.2%]). First, we estimated unadjusted odds ratios using logistic regression models with only the appropriate GP5 variable included. Then, we adjusted for covariates, including treatment age (entered as continuous), baseline ECOG performance status reading (reference, 0), sex (reference, woman), and race (reference, White). Exceptions to entered covariates were the exclusion of sex from the E1Z03 and E1Z11 analyses because these trials included only women. In addition, ECOG performance status readings for E1Z03 were not available.

## RESULTS

Analysis sample sizes, defined as the number of patients who had evaluable GP5 data at any of the included timepoints, for each of the trials were as follows: E1Z03, *N* = 686; E1Z11, *N* = 1026; E1609, *N* = 560; and E1912, *N* = 510. At 3 months, the following numbers of patients completed the GP5 item: E1Z03, *N* = 640; E1Z11, *N* = 916; E1609, *N* = 215; and E1912, *N* = 448. Likewise, at 6 months, the following numbers of patients completed the GP5 item: E1Z03, *N* = 629; E1Z11, *N* = 838; E1609, *N* = 150; and E1912, *N* = 416. Characteristics of the patients included from each trial are listed in Table 1. For each trial, the largest proportion of patients had an ECOG performance status of 0 (excluding E1Z03 because this variable was missing), although a relatively larger proportion of patients in E1912 had an ECOG performance status of 1 (34.7%). The preponderance of participants in E1Z03, E1609, and E1912 were White, exceeding 90% in each trial, although larger proportions of patients in E1Z11 were not White relative to the other trials. Relatively low proportions of patients in E1Z03 and E1Z11 discontinued treatment early due to AEs (5.2% and 4.1%, respectively), whereas 31.6% (*n* = 177) in of patients in E1609 and 16.5% (*n* = 84) of those in E1912 discontinued due to AEs.

**TABLE 1:** Patient characteristics.

	ECOG‐ACRIN trial: No. (%)			
Characteristic	E1Z03, *N* = 686	E1Z11, *N* = 1026	E1609, *N* = 560	E1912, *N* = 510
Baseline ECOG performance status				
0	—	864 (84.3)	479 (85.5)	321 (62.9)
1	—	158 (15.4)	79 (14.1)	177 (34.7)
2	—	3 (0.3)	0 (0.0)	12 (2.4)
3	—	0 (0.0)	0 (0.0)	0 (0.0)
Age: Mean ± SD [range], years	65.0 ± 9.0 [32.0–90.0]	64.0 ± 9.0 [31.0–93.0]	52.0 ± 13.1 [18.0–82.0]	57.0 ± 7.5 [28.0–70.0]
Sex				
Female	686 (100.0)	1026 (100.0)	212 (37.9)	165 (32.4)
Male	0 (0.0)	0 (0.0)	348 (62.1)	345 (67.7)
Race				
American Indian or Alaska Native/unknown	3 (0.4)	0 (0.0)	3 (0.5)	8 (1.6)
Asian/Native Hawaiian or other Pacific Islander	3 (0.4)	204 (19.9)	4 (0.7)	7 (1.4)
Black/African American	22 (3.2)	193 (18.8)	0 (0.0)	29 (5.7)
White	658 (95.9)	629 (61.3)	553 (98.8)	462 (91.3)
Reason for early discontinuation				
Adverse event	36 (5.2)	42 (4.1)	177 (31.6)	84 (16.5)
Disease progression	12 (1.7)	5 (0.5)	119 (21.3)	27 (5.3)
Patient withdrawal	20 (2.9)	57 (5.6)	46 (8.2)	22 (4.3)
Alternative therapy	0 (0.0)	0 (0.0)	1 (0.2)	1 (0.2)
Death	7 (1.0)	0 (0.0)	3 (0.5)	6 (1.2)
Other complicating disease	9 (1.3)	114 (11.1)	1 (0.2)	9 (1.8)
Other	7 (1.0)	35 (3.4)	21 (3.8)	20 (3.9)

Abbreviations: ECOG, Eastern Cooperative Oncology Group; ECOG‐ACRIN, Eastern Cooperative Oncology Group–American College of Radiology Imaging Network; SD, standard deviation.

**FIGURE 1:**
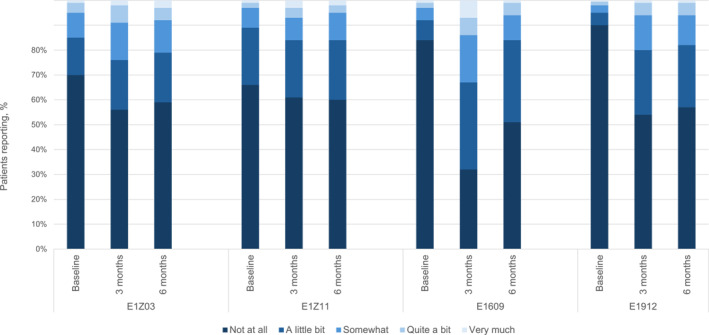
Frequency of GP5 response by trial and timepoint. GP5 indicates item GP5 from the Functional Assessment of Cancer Treatment system (“I am bothered by side effects of treatment”).

Distributions of GP5 responses at each analysis timepoint (baseline, 3 months, and 6 months) are illustrated in Figure 1. Proportions reporting “not at all” on GP5 at baseline were lower in E1Z03 (70.0%) and E1Z11 (66.1%) than in E1609 (84.1%) and E1912 (89.8%). These proportions decreased at 3 and 6 months (E1Z03, 55.6% and 58.8%, respectively; E1Z11, 61.4% and 60.3%, respectively; E1609, 32.6% and 50.7%, respectively; E1912, 54.2% and 56.7%, respectively). The next most frequently endorsed category in each trial and each timepoint was “a little bit.” E1609 and E1912 had the largest proportions reporting moderate‐to‐severe bother (from “somewhat” to “very much”) at 3 and 6 months (E1609, 32.6% and 16.0%, respectively; E1912, 19.7% and 18.8%, respectively).

Table 2 lists the results of the logistic regression analyses estimating odds of early discontinuation due to AEs for patients who reported severe bother (“very much” and “quite a bit”) versus moderate‐to‐low bother (from “somewhat” to “not at all”). The odds ratios were positive (range, 2.59–6.77) and statistically significant (95% CIs did not exceed 1) for all associations at 3 months, including both unadjusted and adjusted models. The largest associations were observed for E1609 (unadjusted odds ratio, 6.74 [95% CI, 2.91–15.64]; adjusted odds ratio, 6.77 [95% CI, 2.88–15.92]). Associations decreased in magnitude at 6 months in each case. The 6‐month associations were statistically significant only for the adjusted model in E1Z03 and for both unadjusted and adjusted models in E1912. Table 3 lists results of the logistic regression analyses examining associations between early discontinuation because of AEs and reporting severe‐to‐moderate bother (from “very much” to “somewhat”) versus low bother (“a little bit” and “not at all”) on the GP5. Compared with the analyses of severe versus moderate/low bother, these effects were always of lower magnitude (3‐month odds ratio: range, 1.90–4.23; 6‐month odds ratio: range, 1.62–3.73) and less often statistically significant.

**TABLE 2:** Odds of early discontinuation due to adverse events for patients rating severe versus moderate‐to‐low bother on item GP5.^a^

ECOG‐ACRIN trial	3‐month OR (95% CI)	6‐month OR (95% CI)
E1Z03^b^		
Unadjusted model	2.59 (1.01–6.61)	2.73 (0.99–7.57)
Adjusted model^c^	2.82 (1.10–7.28)	3.37 (1.20–9.49)
E1Z11^d^		
Unadjusted model	3.84 (1.60–9.24)	NE
Adjusted model^c^	4.10 (1.69–10.00)	NE
E1609^e^		
Unadjusted model	6.74 (2.91–15.64)	3.97 (0.89–17.65)
Adjusted model^c^	6.77 (2.88–15.92)	4.59 (0.86–24.59)
E1912^f^		
Unadjusted model	4.39 (1.74–11.05)	3.28 (1.13–9.46)
Adjusted model^c^	4.15 (1.64–10.49)	3.67 (1.24–10.92)

*Note*: All models exclude patients with a GP5 response of “very much” at baseline.

Abbreviations: CI, confidence interval; ECOG‐ACRIN, Eastern Cooperative Oncology Group–American College of Radiology Imaging Network; GP5, item GP5 on the Functional Assessment of Cancer Treatment (“I am bothered by side effects of treatment”); NE, not estimable (because of a low number of events); OR, odds ratio.

^a^ Severe bother indicates “very much” or “quite a bit”; moderate/low bother, “somewhat,” “a little bit,” or “not at all.”

^b^ Sample size for 3 months, *n* = 640; for 6 months, *n* = 629; 32 patients discontinued early because of adverse events (AEs) for the 3‐month analysis, and 28 discontinued early because of AEs for the 6‐month analysis.

^c^ Adjusts for age (entered as continuous), baseline Eastern Cooperative Oncology Group performance status (reference, 0), sex (reference, female), and race (reference, White).

^d^ Sample size for 3 months, *n* = 891; for 6 months, *n* = 816; 36 patients discontinued early because of AEs for the 3‐month analysis, and 23 discontinued early for the 6‐month analysis.

^e^ Sample size for 3 months, *n* = 215; for 6 months, *n* = 150; 56 patients discontinued early because of AEs for the 3‐month analysis, and 20 discontinued early for the 6‐month analysis.

^f^ Sample size for 3 months, *n* = 448; for 6 months, *n* = 416; 62 patients discontinued early because of AE for the 3‐month analysis, and 38 discontinued early for the 6‐month analysis.

**TABLE 3:** Odds of early discontinuation due to adverse events for patients rating “severe to moderate” versus “low” bother on item GP5.^a^

ECOG‐ACRIN trial	3‐month OR (95% CI)	6‐month OR (95% CI)
E1Z03^b^		
Unadjusted model	2.59 (1.01–6.61)	2.73 (0.99–7.57)
Adjusted model^c^	2.82 (1.10–7.28)	3.73 (1.20–9.49)
E1Z11^d^		
Unadjusted model	4.02 (1.98–8.18)	1.62 (0.58–4.50)
Adjusted model^c^	4.23 (2.06–8.70)	1.82 (0.65–5.12)
E1609^e^		
Unadjusted model^c^	3.78 (1.94–7.36)	2.92 (0.72–7.30)
Adjusted model	3.80 (1.94–7.43)	2.68 (0.78–9.19)
E1912^f^		
Unadjusted model	1.90 (0.98–3.66)	2.33 (1.05–5.17)
Adjusted model^c^	2.02 (1.03–3.97)	2.49 (1.06–5.47)

*Note*: All models exclude patients with a GP5 response of “very much” at baseline.

Abbreviations: CI, confidence interval; ECOG‐ACRIN, Eastern Cooperative Oncology Group–American College of Radiology Imaging Network; GP5, item GP5 on the Functional Assessment of Cancer Treatment (“I am bothered by side effects of treatment”); OR, odds ratio.

^a^ Severe‐to‐moderate bother indicates “very much”, “quite a bit,” or “somewhat”; low bother, “a little bit” or “not at all.”

^b^ Sample size for 3 months, *n* = 640; for 6 months, *n* = 629; six patients discontinued early because of adverse events (AEs) for the 3‐month analysis, and five discontinued early because of AEs for the 3‐month analysis.

^c^ Adjusts for age (entered as continuous), baseline Eastern Cooperative Oncology Group performance status (reference, 0), sex (reference, female), and race (reference, White).

^d^ Sample size for 3 months, *n* = 891; for 6 months, *n* = 816; seven patients discontinued early because of AEs for the 3‐month analysis, and one discontinued early for the 6‐month analysis.

^e^ Sample size for 3 months, *n* = 215; for 6 months, *n* = 150; 56 patients discontinued early because of AEs for the 3‐month analysis, and five discontinued early for the 6‐month analysis.

^f^ Sample size for 3 months, *n* = 448; for 6 months, *n* = 416.

## DISCUSSION

Across four clinical trials for the treatment of breast cancer, melanoma, and chronic lymphocytic leukemia, the FACT GP5 item demonstrated value as an indicator of treatment tolerability through strong associations with early treatment discontinuation due to AEs, even after adjusting for key patient factors. Because the strongest associations were at 3 months postbaseline and often indicated more than two times greater odds of early discontinuation for patients reporting severe side‐effect bother on GP5, this item may be very useful as an early indicator of intolerability. This evidence adds to the growing body of evidence in support of the usefulness of item GP5 across a diverse range of cancer and treatment types.^6^, ^7^, ^9^, ^10^, ^12^

The growing movement to assess tolerability from the patient’s perspective within cancer trials has pointed to the need for end points around overall side‐effect impact.^3^ Recently, two estimands for overall side‐effect impact using GP5 were published: one examining the proportion of patients in severe bother on GP5 in each treatment arm and one examining the number of treatment cycles in severe bother on GP5 in each treatment arm.^18^ A recent FDA labeling claim for selpercatinib in RET‐mutant medullary thyroid cancer used GP5 in a similar approach to the second estimand described above (time in severe bother).^19^ The results presented here provide assurance that differences in the severity of overall side‐effect impact, as captured by GP5, represent differences in the ability to tolerate treatment, as indicated by the odds of early discontinuation, especially when assessed earlier in the trial. This amounts to a type of criterion validity for GP5 as a tolerability end point.

These results also help inform implementation of the GP5 item in future trials by demonstrating differences in results, depending on how the GP5 is operationalized. For three of the four trials examined here (E1Z11, E1609, and E1912), the magnitude of associations was stronger for GP5 dichotomized as severe bother (“quite a bit” and “very much”) versus moderate/low bother (from “somewhat” to “not at all”) compared with severe/moderate bother (from “quite a bit” to “somewhat”) versus low bother (“a little bit” and “not at all”). A previous study examined the association of severe versus low/moderate bother on GP5 with early treatment discontinuation because of AEs in a multiple myeloma trial and observed associations of comparable magnitude to those observed in our current study at 3 months (odds ratio, 3.93; 95% CI: 2.38–6.49).^12^ Conversely, the current study also indicated that severe/moderate bother was significantly associated with early treatment discontinuation, although at a lower magnitude, indicating that selection of the “somewhat” response to GP5 may reflect a clinically salient impact on tolerability for some patients. This finding complements previous evidence that even low‐grade AEs are associated with side‐effect bother.^20^ A reasonable approach in response to our results may be to use the severe versus moderate/low bother dichotomization when the research objective suggests that a comparison of groups at a higher tolerability threshold is most relevant and to use the severe/moderate versus low dichotomization when there is an interest in the less severe but still bothersome side‐effect impacts.

This study's findings call attention to the differential ability for patients to tolerate the same levels of side‐effect severity, often based on factors within the patient’s life that are not related to the treatment itself.^21^, ^22^ Considering interpretation of the “somewhat” category for GP5, it is probable that some patients are better able to tolerate being somewhat bothered by their treatment side effects, whereas others are not. An additional factor is consideration of how much time some patients may be able to tolerate different levels of side‐effect bother; e.g., moderate side effects may be tolerable for short periods of time only for some individuals. We also call attention to the generally lower magnitude of odds ratios at the 6‐month timepoint compared with the 3‐month timepoint for some trials. It is possible that patients who stayed on treatment to the 6‐month timepoints were better able to tolerate side effects than those who discontinued by the 3‐month timepoints. The current analyses were not able to answer these questions, and they should be the subject of future analyses.

This study has both strengths and limitations to consider. The use of four trials that include varying cancer types helps to generalize the utility of item GP5. However, it would be helpful to examine the performance of GP5 in still more cancer treatment settings. Also, whereas important confounders that could be associated with probability for discontinuation were included as covariates in adjusted logistic regression models (e.g., ECOG performance status, age), other factors not captured in these trials might be informative. In particular, we note that some factors that were not adjusted for in this study may directly inform the propensity to stay on treatment given AEs, such as expected length of life or prognosis. Future studies should examine whether these factors may add insight into patients’ treatment tolerability, perhaps when added as an interaction term with GP5 in regression models. This limitation relates to a more general caution about secondary analyses of clinical trial data because these trials were not designed to address the question posed in the current work. Specifically, we note that the numbers of individuals coded as discontinuing treatment because of AE were relatively small, which contributed to wide confidence intervals around some of the odds ratios. Moreover, it is possible that some patients who discontinued due to AEs were coded as discontinued for another reason (e.g., withdrawal). The results should be interpreted with some caution because of these limitations. Nonetheless, these trial data sets provided a unique opportunity to examine how patient‐reported side‐effect impact relates to a well accepted indicator of treatment tolerability: early treatment discontinuation.

In summary, the current study demonstrated the usefulness of the GP5 item in assessing treatment tolerability among patients with different cancer types and on different treatments. The findings indicated a consistent association of patients reporting severe or moderate bother on the GP5 item with a higher likelihood of discontinuing treatment early. This suggests that item GP5 could serve as a valuable and concise tool for monitoring treatment tolerability in patients with cancer and for identifying those at risk of early discontinuation.

## AUTHOR CONTRIBUTIONS

**John Devin Peipert**: Conceptualization; methodology; investigation; writing—original draft; writing—review and editing. **Shaili Ganatra**: Formal analysis. **Fengmin Zhao**: Formal analysis; writing—review and editing. **Ju‐Whei Lee**: Writing—review and editing. **Jessica Roydhouse**: Writing—review and editing. **Edward H. Ip**: Writing—review and editing. **Nathaniel O'Connell**: Writing—review and editing. **Ruth C. Carlos**: Writing—review and editing. **Noah Graham**: Writing—review and editing. **Mary Lou Smith**: Writing—review and editing. **Ilana F. Gareen**: Writing—review and editing. **Ahmad A. Tarhini**: Writing—review and editing. **Tait Shanafelt**: Writing—review and editing. **Vered Sterns**: Writing—review and editing. **Pamela J. Raper**: Writing—review and editing. **David Cella**: Conceptualization, methodology, investigation, writing—review and editing; funding acquisition. **Robert Gray**: Writing—review and editing; funding acquisition. **Lynne I. Wagner**: Conceptualization; investigation; writing—review and editing.

## CONFLICT OF INTEREST STATEMENT

Tait Shanafelt reports grants/contracts from AbbVie, Genentech, and Pharmacyclics LLC outside the submitted work. Vered Sterns reports personal/consulting fees from Novartis and data and safety monitoring for AstraZeneca outside the submitted work. David Cella reports personal/consulting fees from AbbVie, Alexion Pharmaceuticals, Astella Pharma, Bristol Myers Squibb Company, Eisai, GlaxoSmithKline, Novartis, Pfizer Rafael Pharma, Sanofi US Services Inc., and Sermonix outside the submitted work. Robert Gray reports grants/contracts from Exact Sciences outside the submitted work. Lynne I. Wagner reports personal/consulting fees from Celgene Corporation outside the submitted work. The remaining authors declared no conflicts of interest.

## Data Availability

The data that support the findings of this study are available on request from the corresponding author. The data are not publicly available because of privacy or ethical restrictions.
